# Trends in prenatal cares settings: association with medical liability

**DOI:** 10.1186/1471-2458-9-257

**Published:** 2009-07-22

**Authors:** Andrew S Coco, Donna Cohen, Michael A Horst, Angela S Gambler

**Affiliations:** 1 Research Institute & Department of Family and Community Medicine, Lancaster General North Duke Street, Lancaster, PA 17604-3555, USA

## Abstract

**Background:**

Medical liability concerns centered around maternity care have widespread public health implications, as restrictions in physician scope of practice may threaten quality of and access to care in the current climate. The purpose of this study was to examine national trends in prenatal care settings based on medical liability climate.

**Methods:**

Analysis of prenatal visits in the National Ambulatory Medical Care Survey and National Hospital Ambulatory Medical Care Survey, 1997 to 2004 (N = 21,454). To assess changes in rates of prenatal visits over time, we used the linear trend test. Multivariate logistic regression modeling was developed to determine characteristics associated with visits made to hospital outpatient departments.

**Results:**

In regions of the country with high medical liability (N = 11,673), the relative number, or proportion, of all prenatal visits occurring in hospital outpatient departments increased from 11.8% in 1997–1998 to 19.4% in 2003–2004 (p < .001 for trend); the trend for complicated obstetrical visits (N = 3,275) was more pronounced, where the proportion of prenatal visits occurring in hospital outpatient departments almost doubled from 22.7% in 1997–1998 to 41.6% in 2003–2004 (p = .004 for trend). This increase did not occur in regions of the country with low medical liability (N = 9,781) where the proportion of visits occurring in hospital outpatient departments decreased from 13.3% in 1997–1998 to 9.0% in 2003–2004.

**Conclusion:**

There has been a shift in prenatal care from obstetrician's offices to safety net settings in regions of the country with high medical liability. These findings provide strong indirect evidence that the medical liability crisis is affecting patterns of obstetric practice and ultimately patient access to care.

## Background

Medical liability reform remains a high priority issue that continues to receive attention from prominent national organizations, such as the American College of Obstetricians and Gynecologists (ACOG) and the American Medical Association (AMA).[1,2] Medical liability concerns around maternity care have widespread public health implications as restrictions in physician scope of practice may threaten quality of care in the current climate. For example, there is information to show that physician behavior, specifically related to the scope of maternity care, has been altered in areas of high medical liability. A recent survey of high-risk specialist physicians in Pennsylvania showed that 25% of obstetrician/gynecologists often avoided caring for high-risk patients.[3]

Restrictions in scope of practice could lead to a concentration of high-risk patients in safety net settings such as hospital outpatient departments; many of which are already working at full capacity.[4] Previous analysis has shown that visits to hospital outpatient departments are made by sicker patients with less continuity, compared to physicians' offices and community health centers.[5] This combination of excess volume and less continuity in already over taxed hospital systems, particularly among a high-risk patient population, has serious national implications for patient safety and healthcare access issues.

Few studies have quantified the impact of the current malpractice situation on the settings in which prenatal care is delivered at a national level. The purpose of this study is to examine national trends in the settings in which prenatal care occurs based on medical liability climate. Specifically, the aim is to investigate for shifts in the provision of care, including of high-risk patients, from physician's offices to hospital outpatient departments stratified by regions of medical liability. We analyzed data from the National Ambulatory Medical Care Survey (NAMCS) and the National Hospital Ambulatory Medical Care Survey (NHAMCS) from 1997 to 2004.

## Methods

The NAMCS and the NHAMCS are administered by the National Center for Health Statistics (NCHS) for the Centers for Disease Control and Prevention (CDC). [6,7] The surveys are designed to meet the need for objective, reliable information about the provision and use of ambulatory medical care services in the United States. The NAMCS collects information on patient visits to office-based physician practices in the United States, including federally qualified health centers and non-federal government clinics. The NHAMCS collects information on patient visits to non-federal hospital outpatient departments and hospital emergency departments separately. The hospital emergency department data was not utilized in this study. Both surveys incorporate multistage probability designs. NAMCS has a three-tiered design based on geographic location, physician specialty and individual visits within the practice. The NCHS weighs each visit by taking into account practice location and physician specialty. The NHAMCS has a four-tiered sampling design based on geographic area, hospitals within the area, outpatient departments within hospitals, and patient visits. The panel of hospitals for NHAMCS is rotated so that a given hospital is included every 15 months.

Physicians (NAMCS only) are randomly selected from the master files of the American Medical Association and the American Osteopathic Association. Each physician is randomly assigned to a one-week reporting period. During this period, data for a systematic random sample of visits are recorded by the physician or office staff on a standardized encounter form provided for that purpose and checked for completeness by NCHS field staff. Hospitals (NHAMCS only) were sampled for the 2004 NHAMCS from a hospital databases called "Healthcare Market Index" and "Hospital Market Profiling Solution." Hospitals with an average length of stay for all patients of less than 30 days or hospitals whose specialty was general adult or children's general were eligible. Data was collected by hospital staff in the same manner as the NAMCS procedure.

Clinical and demographic data, including insurance status, race, and ethnicity were collected for each visit.[8] Categories of race include White, African American/Black, and Asian/Other; while categories for ethnicity include Latino/Hispanic or Not Latino/Hispanic. All patient demographic information is de-identified to prevent linkage with individual patients. The NCHS institutional review board approved the protocols for the NAMCS and NHAMCS, including a waiver of the requirement for informed consent. Provider and hospital variables include self-selected specialty (NAMCS only), clinic type (NHAMCS only), whether seen by a midlevel provider (nurse practitioner, midwife, or physician assistant), whether seen by a resident physician (NHAMCS only), and geographic region. Clinical variables included the primary diagnosis coded according to the International Classification of Diseases (ICD-9-CM).[9]

The surveys collected 451,710 patient records between 1997 and 2004.[8] The participation rate of contacted physician practices in the NAMCS ranged from 63% in 1999 to 70% in 2002. The participation rate of contacted hospitals in the NHAMCS ranged from 91% in 2004 to 98% in 1998. Quality control was performed using a 2-way independent verification procedure for 10% of the sample records. In 2004, coding errors for various items ranged from 0% to 0.9% in the NAMCS and from 0.1% to 1.5% in the NHAMCS.

The NAMCS and NHAMCS surveys incorporate a multistage probability design to generate a population-based sample, accounting for practice location, outpatient departments within hospitals, physician specialty, and patient visits. The sampling technique and probability design utilized by the National Center for Health Statistics (NCHS) allows extrapolation to national estimates for all aspects of the survey, making our study findings representative of the nation as a whole. The NCHS weights each visit to allow extrapolation to national estimates for all aspects of the surveys. National estimates are considered reliable with a standard error of 30% or less; which generally corresponds to a sample of at least 30 patient visits.[8]

To examine trends in the rates of all national prenatal visits, we examined all office and hospital outpatient visits with the primary diagnoses of normal pregnancy (ICD-9-CM code V22), supervision of high risk pregnancy (ICD-9-CM code V23), or obstetrical complications (ICD-9-CM codes of 640–673). In our categorization of prenatal visits we were aware that these 3-digit ICD-9-CM codes could include diagnoses related to labor, postpartum, or some unspecified aspect of care. Consequently we included only visits in which the fifth digit of the 5-digit ICD-9-CM code was 3, the coded indicator for an antepartum condition or complication. There were 21,454 sample records that met all inclusion and exclusion criteria during the eight year study period. A variable was created to designate complicated prenatal visits by excluding those visits with the diagnosis of normal pregnancy (ICD-9-CM code V22). A total of 5,779 sample records met this criterion.

We included visits made to obstetricians' offices. The National Hospital Ambulatory Medical Care Survey does not include data on visits made to family medicine outpatient departments (rather only general medical clinics where it is unclear the specialty of the provider). Therefore, we only included visits occurring in obstetricians' offices, in order to allow for equitable comparisons and trend analyses in the study.

**Figure 1:**
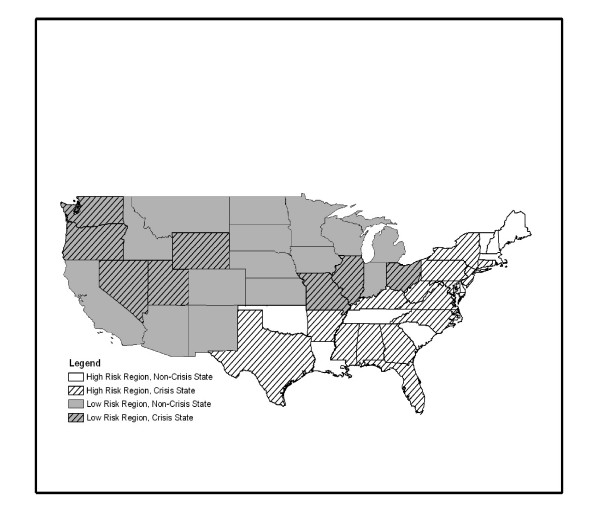
**Medical liability regions with ACOG "Red Alert" or AMA "Crisis" States**. Crisis states were either designated "Red Alert" status by the American College of Obstetricians and Gynecologists or "Crisis" status by the American Medical Association. High medical liability region is comprised of states in the Northeast and South. In 2004, 82% of births in this half of the country occurred in crisis states. Low liability region is comprised of states in the Midwest and West. In 2004, 33% of births in this half of the country occurred in crisis states.

We identified two geographical regions of medical liability, high and low; by utilizing a combination of the ACOG and AMA state medical liability status designations with the geographical region survey item available in NAMCS and NHAMCS. The National Center for Health Statistics (NCHS) divides the country into four geographical regions–Northeast, South, Midwest, and West–in NAMCS and NHAMCS. There is no variable that identifies the individual state in which the visit occurred. At the end of the study period, in 2004, ACOG had designated 22 states "Red Alert" status indicating that liability insurance for the specialty had become so expensive that ob-gyns were restricting or abandoning their obstetrical practices.[1] Similarly, the AMA asserted that 19 states were in a condition of full medical liability "Crisis" in 2004.[2] Seventeen of the 19 AMA "Crisis" states overlapped with the ACOG "Red Alert" states. A total of 24 states met the criteria for at least one of the designations (Figure 1). The annual number of live births for each medical liability region was obtained from National Center for Vital Statistics Reports for 2004 (Table 1).[10] The Northeast and South regions were combined–82% of live births occurred in "Red Alert" or "Crisis" states–and designated the high medical liability region or half of the country. The Midwest and West regions were combined–33% of live births occurred in "Red Alert" or "Crisis" states–and designated as the low medical liability region or half of the country.

**Table 1:** Percentage of live births in malpractice crisis states in high and low medical liability regions in 2004[11]

**High Medical Liability Region**		**Low Medical Liability Region**	
States	Live Births	States	Live Births

**Crisis**		**Crisis**	
			
Alabama	59,510	Illinois	180,778
Arkansas	38,573	Missouri	77,765
Connecticut	42,095	Nevada	35,200
District of Columbia	7,933	North Dakota	8,189
Florida	218,053	Ohio	148,954
Georgia	138,849	Oregon	45,678
Kentucky	55,720	Utah	50,670
Maryland	74,628	Washington	81,747
Mississippi	42,827	Wyoming	6,807
New Jersey	115,253		
New York	249,947	Total crisis state live births	635,788
North Carolina	119,847		
Pennsylvania	144,748	**Non-crisis**	
			
Texas	381,293	Alaska	10,338
Virginia	103,933	Arizona	93,663
West Virginia	20,880	California	544,843
		Colorado	68,503
Total crisis state live births	1,814,089	Hawaii	18,281
		Idaho	22,532
**Non-crisis**		Indiana	87,142
			
Delaware	11,369	Iowa	38,438
Louisiana	65,369	Kansas	39,669
Maine	13,944	Michigan	129,776
Massachusetts	78,484	Minnesota	70,624
New Hampshire	14,565	Montana	11,519
Oklahoma	51,306	Nebraska	26,332
Rhode Island	12,779	New Mexico	28,384
South Carolina	56,590	South Dakota	11,338
Tennessee	79,642	Wisconsin	70,146
Vermont	6,599		
			
Total non-crisis state live births	390,647	Total non-crisis state live births	1,271,528
			
Regional Total	2,204,736	Regional Total	1,907,316
			
% of live births in crisis states (1,814,089/2,204,736)	**82.3%**	% of live births in crisis states (635,788/1,907,316)	**33.3%**

Crisis state = ACOG "Red Alert" or AMA "Crisis" designation.

We calculated standard errors (SEs) for all results as recommended by the NCHS using STATA software, which accounts for the complex sampling design of the NAMCS/NHAMCS.[8] STATA was programmed with the masked survey design variables as recommended by the Ambulatory Statistics Branch of the National Center for Health Statistics.[11] We evaluated categorical variables with the χ^2 ^test. To assess changes in rates of prenatal visits over time, we used the linear trend test. In order to decrease annual variation in visit estimates we combined two successive years of data, as recommended by NCHS, for a total of four two-year periods (1997–1998, 1999–2000, 2001–2002, 2003–2004) for the trend analyses.[8] All statistical trend tests take into account data from all 8 years from 1997 through 2004.

We developed a multivariable logistic regression model adjusting for patient demographic, insurance, continuity, provider, and setting and diagnosis information that included all prenatal visits and had hospital outpatient department as the dependent variable (n = 21,454). This model reflects our hypothesis that prenatal visits in hospital outpatient departments occurred with patients who were minority, more complicated, less established in care and with less insurance. We used the computer program STATA^® ^Intercooled, version 9.0 to analyze all data. All *P *values are 2-tailed; *P*<.05 was considered significant.

## Results

The 21,454 study visits represented an estimated 211 million (95% confidence interval [CI], 191–231 million) prenatal visits for an annual of average of 26 million visits. Overall 13.8% (95% CI, 12.1% – 15.6%) of prenatal visits occurred in hospital outpatient departments and 86.2% of visits took place in obstetrician's offices. In the country as a whole, these proportions did not change over time; hospital outpatient department visits ranged from 12.4% in 1997–1998 to 15.5% in 1999–2000 to 13.7% in 2003–2004 (*P *= .72 for trend). Prenatal visits to FQHCs and non-federal government clinics accounted for 3.6% of all visits to physicians' offices, ranging from 5.2% in 1997–1998 to 3.8% in 2003–2004. However, the sample of visits in 2001–2002 was too small to allow for a trend analysis.

**Figure 2:**
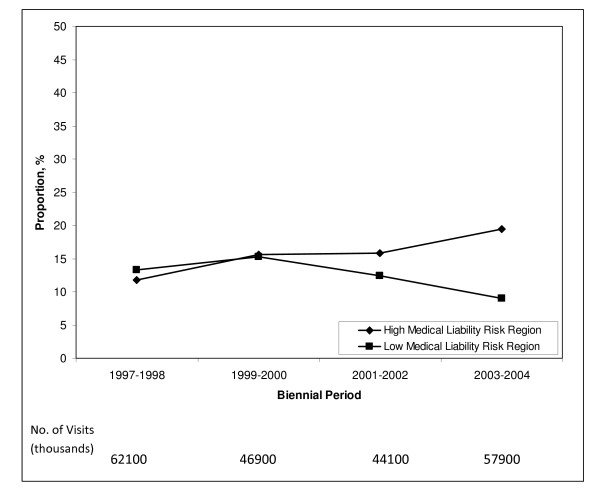
**Proportion of prenatal visits seen in hospital outpatient departments compared to obstetrician's offices in high and low medical liability regions, 1997–2004**. National estimates based on 21,454 visits in the National Ambulatory Medical Care Survey and the National Hospital Ambulatory Medical Care Survey. High medical liability risk region is comprised of states in the Northeast and South. Low medical liability risk region is comprised of states in the Midwest and West. For trend: *P *< .001 for high liability risk region and *P *= .012 for low liability risk region.

The delivery setting for prenatal care shifted in the high medical liability region from obstetrician's offices to hospital outpatient departments. The proportion of prenatal visits (n = 11,673) seen in hospital outpatient departments increased by 64% from 11.8% in 1997–1998 to 19.4% in 2003–2004 (*P *< .001 for trend) (Figure 2). The reverse trend occurred in the low medical liability region, where the proportion of prenatal visits (n = 9,781) seen in hospital outpatient departments decreased by 32% from 13.3% in 1997–1998 to 15.3% in 1999–2000 to 12.4% in 2001–2002 to 9.0% in 2003–2004 (*P *= .012 for trend) (Figure 2).

**Figure 3:**
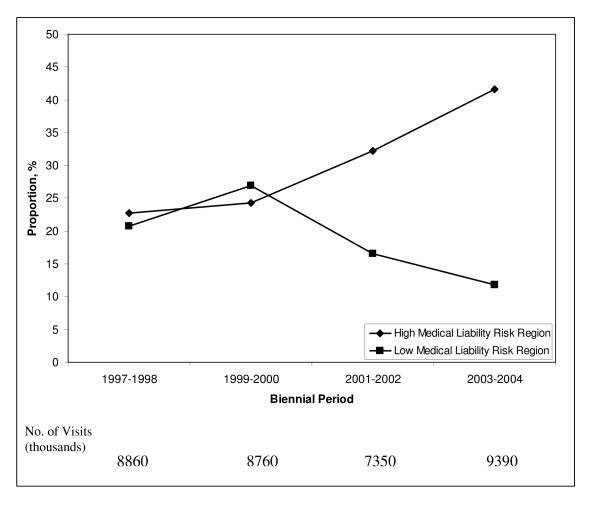
**Proportion of visits with a complicated prenatal diagnosis seen in hospital outpatient departments compared to obstetrician's offices in high and low medical legal risk regions, 1997–2004**. National estimates based on 5,779 visits in the National Ambulatory Medical Care Survey and the National Hospital Ambulatory Medical Care Survey. High medical liability risk region is comprised of states in the Northeast and South. Low medical liability risk region is comprised of states in the Midwest and West. For trend: *P *= .004 for high liability risk region and *P *= .04 for low liability risk region.

Further analysis for trends was performed when visits were restricted to only complicated prenatal visits (n = 5,779). The overall proportion of visits with a complicating diagnosis was 16.3% (95% CI, 14.2% – 18.4%). The proportion of complicated prenatal visits seen in hospital outpatient departments, again, showed that visits were being shifted to this safety net setting from obstetrician's offices in the high medical liability region but not in the low medical liability region. In the high medical liability region, the proportion of all complicated visits (n = 3,275) seen in hospital outpatient departments increased by 83% from 22.7% in 1997–1998 to 41.6% in 2003–2004 and reciprocally decreased in obstetrician's offices from 77.3% to 58.4% over the same period (*P *= .004 for trend) (Figure 3). In the low medical liability region the proportion of complicated visits (n = 2,504) seen in hospital outpatient departments decreased by 43% from 20.8% in 1997–1998 to 26.9% in 1999–2000 to 11.8% in 2003–2004 (P = .04 for trend) (Figure 3). The proportion of routine prenatal visits (ICD-9-CM code, V22) occurring in hospital outpatient departments (n = 8,398) also increased, although less dramatically, in the high medical liability region from 9.9% in 1997–1998 to 14.9% in 2003–2004 (*P *= .02 for trend). In the low medical liability region the proportion of routine prenatal visits (n = 7,277) in the hospital outpatient department showed a non significant decrease from 12.1% in 1997–1998 to 12.8% in 1999–2000 to 8.5% in 2003–2004 (*P *= .07 for trend).

The proportion of all ambulatory visits (n = 451,013), not just prenatal visits, that occurred in hospital outpatient departments was analyzed as a means of assessing regional changes in the capacity of this component of the safety net setting. The proportion remained stable over the study period in the both regions varying from 9.5% in 1997–1998 to 10.7% in 1999–2000 to 9.1% in 2001–2002 to 9.4% in 2003–2004 in the high medical liability region and from 7.5% in 1997–1998 to 8.4% in 2003–2004 in the low liability region.

In multivariable logistic regression modeling, independent predictors of a hospital outpatient department visit versus an obstetrician's office visit were Latino ethnicity (vs non-Latino ethnicity: odds ratio [OR], 1.76; 95% CI, 1.28 – 2.42), African American race (vs White race: OR, 2.87; 95% CI, 2.04 – 4.05), Asian or other race (vs White race: OR, 1.67; 95% CI, 1.07–2.61), Medicaid insurance (vs private insurance: OR, 6.06; 95% CI, 4.40 – 8.34), self-pay (vs private insurance: OR, 11.08; 95% CI, 6.74 – 18.22), other insurance (vs private insurance: OR, 4.97; 95% CI, 3.20 – 7.73), with a complicating diagnosis (vs without a complicating diagnosis: OR, 1.69; 95% CI, 1.21 – 2.37), with a new patient (vs with an established patient: OR, 1.86; 95% CI, 1.36 – 2.55), and seen by a midlevel provider (vs not seen by a midlevel provider: OR, 3.79; 95% CI, 2.07–6.94). (Table 2) Visit characteristics were similar between high and low medical liability regions except for racial make-up (Table 3).

**Table 2:** Prenatal visit characteristics of hospital outpatient departments and obstetrician's offices, 1997–2004 (n = 21,454)

Characteristic	Proportion of visits in Hospital outpatient departments, %	Proportion of visits in Obstetrician's offices, %	Adjusted* OR with (95% CI)
Age, years			
< 23	38	22	1.00
23–28	31	32	.91 (.76 – 1.10)
> 28	31	46	.89 (.73 – 1.07)

Latino ethnicity	22	12	1.76 (1.28 – 2.42)

Race			
White	61	83	1.00
African American	33	12	2.87 (2.04 – 4.05)
Asian and other	6	5	1.67 (1.07 – 2.61)

Insurance			
Private	22	69	1.00
Medicaid	56	21	6.06 (4.40 – 8.34)
Self-pay	10	3	11.08 (6.74 – 18.22)
Other	12	7	4.97 (3.20 – 7.73)

Obstetrical Complication	24	12	1.69 (1.21 – 2.37)

New patient	11	5	1.86 (1.36 – 2.55)

Abbreviations: OR, odds ratio; CI, confidence interval.

Dependent variable: hospital outpatient department visit.

*Odds Ratio adjusted for all other listed variables.

## Discussion

This study used two nationally representative surveys to characterize and contrast prenatal visits made to hospital outpatient departments and physician offices in areas of varying medical liability. The high medical liability region, represented by 25 states and the District of Columbia in the Northeast and South, experienced a significant migration of patients from physicians' offices to hospital outpatient departments, a traditional safety net delivery site. If the trend continues an annual estimate of 1.36 million visits would shift from these settings. Among complicated prenatal visits this trend was even more striking. The results of our analysis provide strong indirect evidence that the medical liability crisis is affecting patterns of obstetric practice and ultimately patient access to care.

Our analysis has several limitations that should be considered when interpreting the data on prenatal visits. First, due to limitations in the data, we were unable to perform a direct comparison of prenatal visit rates between states with and without an adverse malpractice climate. Instead, states within one of four geographical regions were combined into one of two national regions based on the percentage of births that occurred in an adverse malpractice climate. Over 82% of the births in the high medical liability region occurred in states considered to be in medical malpractice "crisis" by ACOG or the AMA compared with 33% in the low medical liability region. However, the effect of this crossover, if any, would be to bias the results against the trends we have shown. That is, a direct comparison of states with and without an adverse medical malpractice climate might have shown more pronounced trends in prenatal visit rates than was determined in our study.

Second, prenatal visits made to community health centers, which are considered to be a major component of the US safety net, were too small to allow for trend analysis in these databases. Forrest and Whelan demonstrated that in 1994 "obstetric services for pregnant women comprised 5.8% of primary care visits made to community health centers", totaling approximately 757 visits, compared to 26,067 visits made to physician offices and 2,587 to hospital outpatient departments.[5] Exclusion of these visits to community health centers likely represents a relatively small proportion of the total national visits, especially given the stable proportions of prenatal visits seen in both obstetrician's offices and hospital outpatient departments over the 8 year period in this study. Moreover, exclusion of community health center visits does not detract from the trend seen in hospital outpatient departments located in high medical liability regions, but may actually represent a potentially larger safety net population affected by changes in physician practice.

Additionally, we lacked direct data on the capacity of hospital outpatient departments between 1997 and 2004, which may have accounted for the increased proportion of visits in the high medical liability region. We therefore analyzed all ambulatory visits made to hospital outpatient departments as a proxy for differences in the capacity of the safety net setting among medical liability regions. Our analysis confirmed that the increased prenatal visits were not secondary to an increased number of hospital departments but more so a result of physicians restricting scope of practice when concerned about medical liability.

Finally, there were other variables for which our multivariate logistic regression model was unable to control that might impact our study findings. In some regions, particularly in high medical liability regions where the cost of medical liability insurance may be prohibitive, obstetrical providers might be driven to sell their practices to hospitals and therefore become employees of the hospital department. Changes in the workforce, such as increased women entering obstetrics, and preferences for the balance between lifestyle and work may also influence some providers to move towards working for hospital outpatient departments rather than dealing with some of the pressures associated with private practice. In summary, there may be other factors impacting the described shift to hospital outpatient departments for which our study was not able to account.

While studies validate that there are concerns over the ability of the safety net to provide adequate access to care to a population that likely includes sicker, higher risk patients, it is true that maternal-fetal medicine specialists, as well as experienced obstetricians involved with teaching programs for example, tend to be hospital based providers. Unfortunately, the database is unable to resolve this issue, as only a small proportion of visits within hospital outpatient departments were categorized as visits made to maternal-fetal medicine specialists. Although these visits may represent improved care being delivered to this subset of patients, it is unlikely that the entire increasing trend represents a shift of patients fully being seen by maternal-fetal medicine. In addition, we would still expect that many of the conditions documented in previous studies (poor access, limited funding, insurance coverage, patient population with more complex social-medical issues) are associated with this increased shift of visits to hospital outpatient departments. Lastly, as mentioned above, there was an equal proportion of complicated obstetrical visits in low and high medical liability regions (16% vs 16%, p = 0.99). Therefore, one would expect the shift of visits being made to maternal-fetal medicine specialists to be equally distributed between low and high medical liability regions.

The sample size of 21,454 patient visits and 5,799 complicated visits was representative of approximately 211 million prenatal visits for the 10-year period. By utilizing the sampling and probability techniques discussed in the Methods section, the data was reflective of ambulatory visits made nationally. Within the database, an individual patient could not be tracked over the study period. Theoretically, if a patient made more than one visit to the office or hospital outpatient department within the one-week period that sampling occurred, then a patient may have been counted more than once. One would expect multiple visits within a one-week period to occur for complicated obstetrical patients. However, Table 3 (pg 20) demonstrates that an equal proportion of complicated obstetrical visits occurred in low and high medical liability regions (16% vs 16%, p = 0.99). Therefore, even if a patient visit was counted more than once, given the equal distribution of complicated obstetrical visits among low and high medical liability regions, one would not expect this to impact the study findings.

**Table 3:** Prenatal visit characteristics of hospital outpatient departments and obstetrician's offices: comparison of low and high medical liability regions (n = 21,454)

Visit Characteristic	Low Medical liability region, % of visits	High Medical liability region, % of visits	p value
Age, years			

< 23	23	25	.18
	
23–28	34	30	
	
> 28	43	45	

			

Latino ethnicity	16	18	.42

			

Race			

White	82	78	< .01
	
African American	10	19	
	
Asian and other	8	3	

			

Insurance			

Private	64	61	.06
	
Medicaid	25	27	
	
Self-pay	2	5	
	
Other	8	7	

			

Obstetrical Complication	16	16	.99

			

New patient	7	6	.41

			

Non-MSA location	11	13	.54

			

Midlevel Provider	5	6	.67

			

Resident Provider	36	27	.10

Despite these limitations, there were strong trends in prenatal care shifting to the hospital outpatient department from physicians' offices in the high medical liability region. Over the study period, the proportion of prenatal visits made to hospital outpatient departments increased significantly from 1 in 8 (12%) in 1997–1998 to almost 1 in 5 (19%) in 2003–2004. The reverse shift occurred in the low medical liability region, and once again supports our findings that in areas where medical liability is of concern, prenatal care is being made less available in physicians' offices and consequently being shifted to safety net settings. Hospital outpatient visits were more likely to be associated with no insurance, Medicaid insurance, obstetrical complications, non-established patients, non-white race, and Latino ethnicity.

Within the high medical liability region, the shift of visits from obstetrician's offices to hospital outpatient departments occurred at an even more striking rate for prenatal visits with obstetrical complications. The concurrent shift in routine prenatal visits, combined with the opposite trend in the low liability region, argues against the shift for complicated patients resulting from referrals for specialty care only available in hospital outpatient departments. Rather, this consistent shift of care to safety net settings, particularly among visits with a medically complicated diagnosis, is worrisome for a system already strained by financial, economic, and provider considerations. If the described trends in prenatal care continue, patients in the high medical liability half of the country will undoubtedly experience increased difficulty with access to and use of prenatal care. This is of particular concern for patients with complex, high-risk diagnoses during pregnancy who likely require increased surveillance, monitoring, and access to their physicians during pregnancy.

In low medical liability regions, the assumption from our findings is that in regions of low risk medical liability, obstetricians felt more comfortable continuing to provide care to patients. This study is based on the cross-sectional designation of medical liability by the ACOG and AMA in 2004. Therefore we do not have available data on the possible medical liability climate prior to the 10-year period in this analysis. One hypothesis for the trend out of hospital outpatient departments into physicians' offices in low medical liability regions might be that if these regions had experienced an improvement in medical liability practice climate compared to 1995 (for example), then one might see a shift from hospital outpatient departments into physicians' offices as seen in our study. The current study does not have available data on the medical liability climate prior to 2004 but this may be a point of interest for future analyses.

The findings from this study have widespread public health and policy implications. The reallocation of patients, particularly complicated patients, to hospital outpatient departments is evidence of the increasing burden being placed on our nation's health care safety net. Lurie comments that "the safety net ideally should go beyond simply providing access to provide high-quality care to those it serves" and highlights the concerns over the ability of the safety net to meet the increasing demands of providing access to care and remain a viable entity.[12] In 2000, the Institute of Medicine identified three obstacles that providers within the safety net face: 1) the expanding numbers of uninsured persons; 2) the threat to the traditional sources of funding; and 3) the shift to Medicaid managed care.[12,13] Previous analysis has shown that visits to hospital outpatient departments are made by sicker patients with less continuity compared to physician's offices and community health centers.[5] Additionally, care at these sites is more likely to be provided by the practitioners less likely to be trained or experienced with high-risk patients, such as mid-levels or resident physicians. Our analysis validates these concerns and demonstrates the increasing burden being placed on the safety net system, where the quality of care may be diminished due to over taxed institutions, less experienced providers, and most importantly a concentration of patients with more complex medical and social issues.

## Conclusion

The AMA and ACOG both contend that the current medical liability climate is forcing physicians to restrict their practice and therefore limiting patients' access to care. Our study findings provide national data to substantiate this belief. The high medical liability region, represented by 25 states and the District of Columbia in the Northeast and South, experienced a significant migration of patients from physicians' offices to hospital outpatient departments, a traditional safety net delivery site. Among complicated prenatal visits this trend was even more striking, providing strong indirect evidence that the medical liability crisis is affecting patterns of obstetric practice. Liability reform efforts must work to inform policy changes and tort reform that will help to reverse physicians' decisions to limit scope of practice or entry into the specialty, and ultimately improve patients' access to prenatal and obstetric care.

## Competing interests

The authors declare that they have no competing interests.

## Authors' contributions

AC conceived of the study, participated in its design, performed the statistical analysis, interpreted the data, and assisted with manuscript preparation. DC participated in analysis of study results, interpretation of the data, and manuscript preparation. MH performed the statistical analysis and reviewed the manuscript. AA assisted with data collection, statistical analysis, and reviewed the manuscript. All authors read and approved the final manuscript.

## Pre-publication history

The pre-publication history for this paper can be accessed here:


